# Efficient ${\boldsymbol{A}}{\boldsymbol{u}}{({\boldsymbol{C}}{\boldsymbol{N}})}_{{\bf{2}}}^{-{\bf{1}}}$ adsorption using peach stone-derived granular activated carbon

**DOI:** 10.1038/s41598-019-39964-y

**Published:** 2019-03-04

**Authors:** Tayengwa Christopher Maponga, Courtie Mahamadi

**Affiliations:** Chemistry Department, Bindura University of Science Education, P. Bag 1020, Bindura, Zimbabwe

## Abstract

Agricultural wastes have attracted attention as alternative precursors for the production of granular activated carbon. Using batch sorption studies, we demonstrated that granular activated carbon prepared through chemical activation using zinc chloride followed by physical activation in nitrogen environment at high temperatures can effectively adsorb aurocyanide from solution. Furthermore, gold recovery process using the granular activated carbon was amenable to optimization of adsorption parameters such as carbon dosage, initial aurocyanide concentration and contact time. These results may open new fronts on the application of low-cost granular activated carbon, particularly in the carbon-in-pulp metallurgical process.

## Introduction

The recovery of gold from primary sources such as leach solutions and secondary sources such as electronic scrapes and waste electroplating solutions has attracted significant research interest over the years^1–5^. The conventional methods for recovering gold include leaching, ion exchange and adsorption^6^. Comparatively, adsorption seems to be the most suitable method for recovery of precious metals such as gold in the case of low concentration due to its high efficiency and low cost.

Activated carbon, which refers to carbonaceous materials with high porosity, high physicochemical stability, high adsorptive capacity, high mechanical strength, high degree of surface reactivity, and immense surface area has been widely used for the recovery of gold by adsorption of aurocyanide complexes onto activated carbon^6^. This occurs through commercial metallurgical processes which apply counter-current stirred system (the carbon-in-pulp or C.I.P process), in packed adsorption columns or fluidized bed reactors^7–9^. Production of activated carbon can occur through the use of naturally occurring as well as synthetic carbonaceous starting material or precursor, with the quality and properties of the activated carbon strongly depending on the type of precursor^10,11^. It has also been established that the properties of the activated carbon are also influenced by types of activating agents, time, impregnation condition and carbonization temperature^12–14^.

The use of agricultural by-products or wastes as precursors for the production of activated carbon has drawn the attention of many researchers. Through pyrolysis, the waste material can be converted into activated carbon by treatment at varying temperature with or without chemical activating agents^15^. This way, the waste material is converted into useful, relatively low-cost activated carbon, thereby achieving value addition. Recently, several workers have reported on the potential of agricultural waste as precursor for activated carbon. These include materials such as coconut shell^16^, wheat bran^17^, Lignin^18^, chestnut oak shells^19^, Pine Cone^20^, and palm oil wastes^21^.

In this work, the use of peach stones as a precursor for production of granular activated carbon was investigated. This kind of precursor has already been reported in the literature and the activated carbon produced tested for the adsorption of gold. However, our scan of the literature reveal that the activation process has mainly been through pyrolysis with or without steam activation. In some cases the granular activated carbon was tested for the adsorption of gold from thiourea solutions^22^. Thus, the aim of this work was to evaluate the use of granular activated carbon derived from peach stones as an alternative for gold recovery from diluted solutions. More specifically, the study sought to produce granular activated carbon using peach stones as precursor and to determine the effect of different chemical treatment agents and activation temperature on the aurocyanide adsorption properties of the carbon. Kinetic and equilibrium adsorption characteristics of aurocyanide onto the granular activated carbon were evaluated as a function of carbon dosage, initial aurocyanide concentration, and contact time for zinc chloride and phosphoric acid-treated material carbonized at varying temperatures.

## Results

### Characterization

#### Percentage Burn-off

Determination of Burn-off, which is the weight loss of pyrolyzed char determined on a dry weight basis, has been reported previously^23^. The amount of original precursor remaining after pyrolysis and activation gives the yield:

1 $$ \% \,Burn-off=(\frac{(wg{t}_{ba}-wg{t}_{aa})}{wg{t}_{ba}})\times 100$$

and

2 $$ \% \,Yield=(wg{t}_{aa}/wg{t}_{p})\times 100$$

where:

3 $$wg{t}_{ba}={\rm{dry}}\,{\rm{weight}}\,{\rm{before}}\,{\rm{activation}}$$

4 $$wg{t}_{aa}={\rm{dry}}\,{\rm{weight}}\,{\rm{after}}\,{\rm{activation}}$$

5 $$wg{t}_{p}={\rm{dry}}\,{\rm{weight}}\,{\rm{of}}\,{\rm{precursor}}\,{\rm{before}}\,{\rm{pyrolysis}}$$

**Figure 1:**
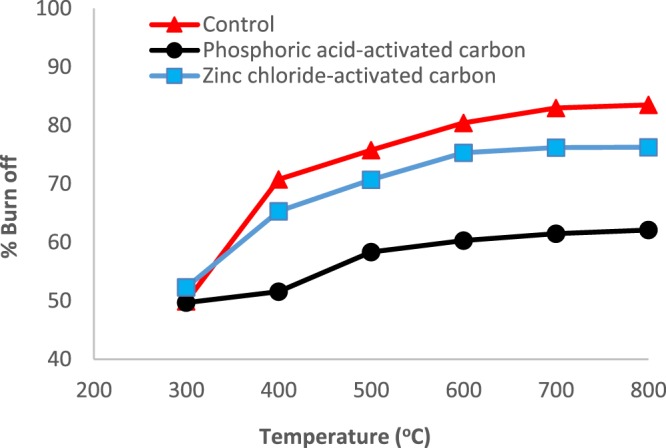
Plots of % Burn-off against temperature (°C) for the control and chemically activated carbons.

The results for the variation of % Burn-off with temperature are shown in Fig. 1. Generally, the % Burn-off increased with temperature, leveling off at higher activation temperatures. Furthermore, it can also be seen that the % Burn-off increased in the order Control > Zinc chloride > Phosphoric acid-activated carbon.

#### Hardness number

**Figure 2:**
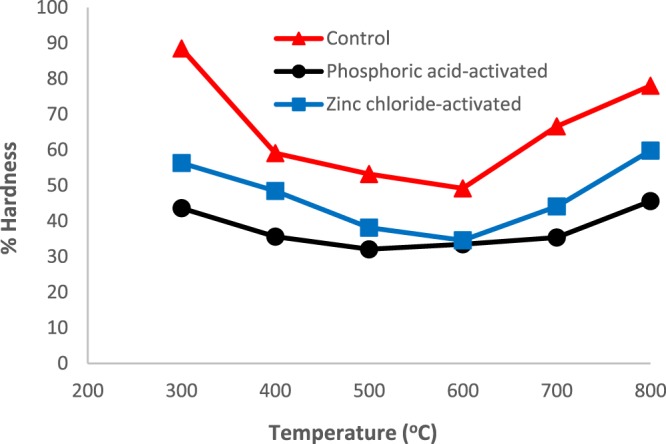
Plots of % Hardness number against Temperature (°C) for the control and chemically activated carbons.

Percentage hardness number gives a measure of resistance to crushing and breakage of granular carbons. This is important for evaluation of potential industrial applications of the carbons as it determines the capacity for regeneration and reuse of the material. Figure 2 shows that the % Hardness number initially decreased with temperature in the range 300–600 °C, before increasing and followed the trend: Control > Zinc chloride-activated > Phosphoric acid-activated carbon.

### Surface characterization

#### SEM characterization

**Figure 3:**
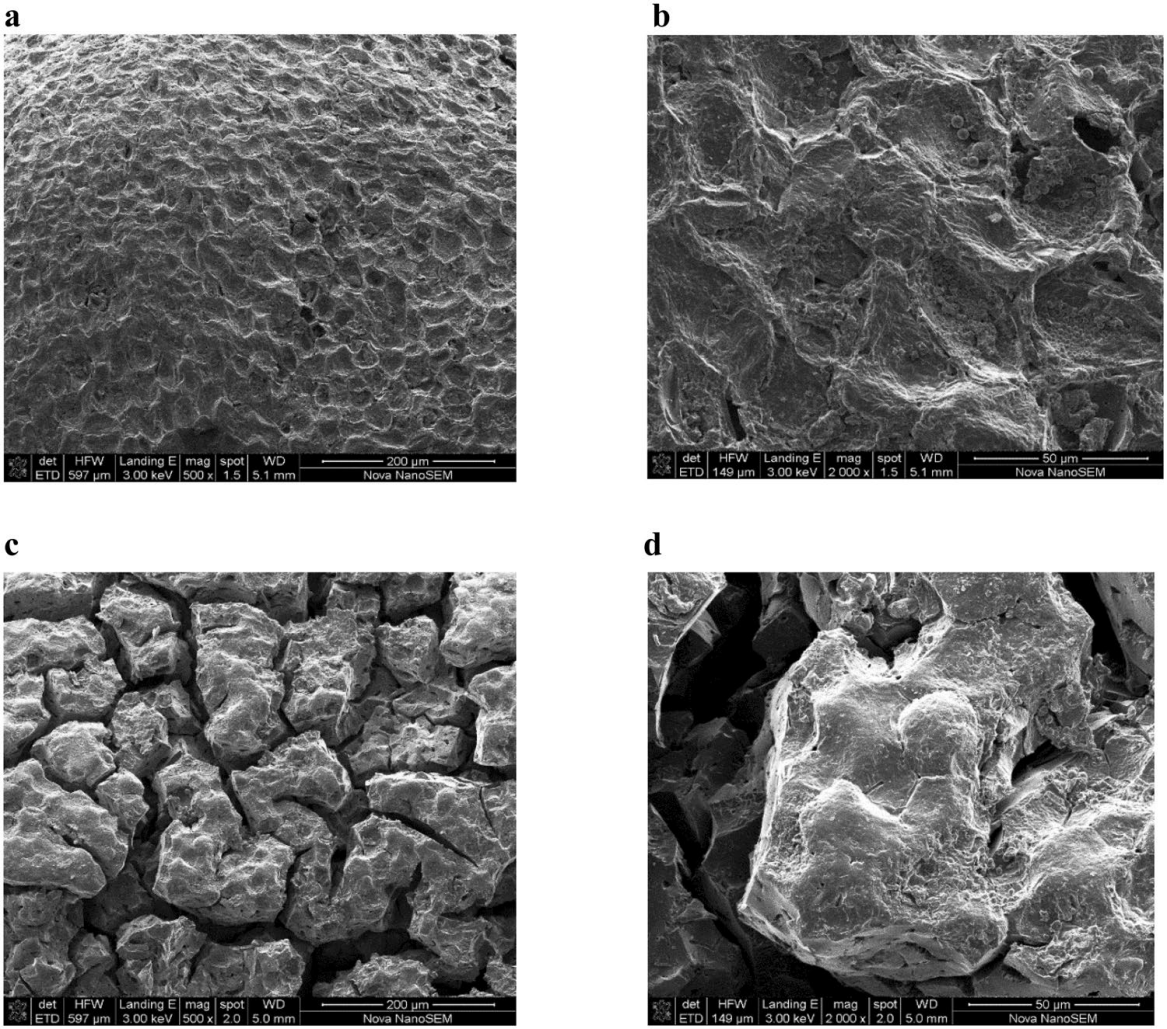
Scanning electron microscopy of H_3_PO_4_-treated carbon activated at 300 °C magnified at (**a**) 500× and (**b**) 2000× respectively and carbon activated at 400 °C, magnified at (**c**) 500× and (**d**) 2000× respectively.

**Figure 4:**
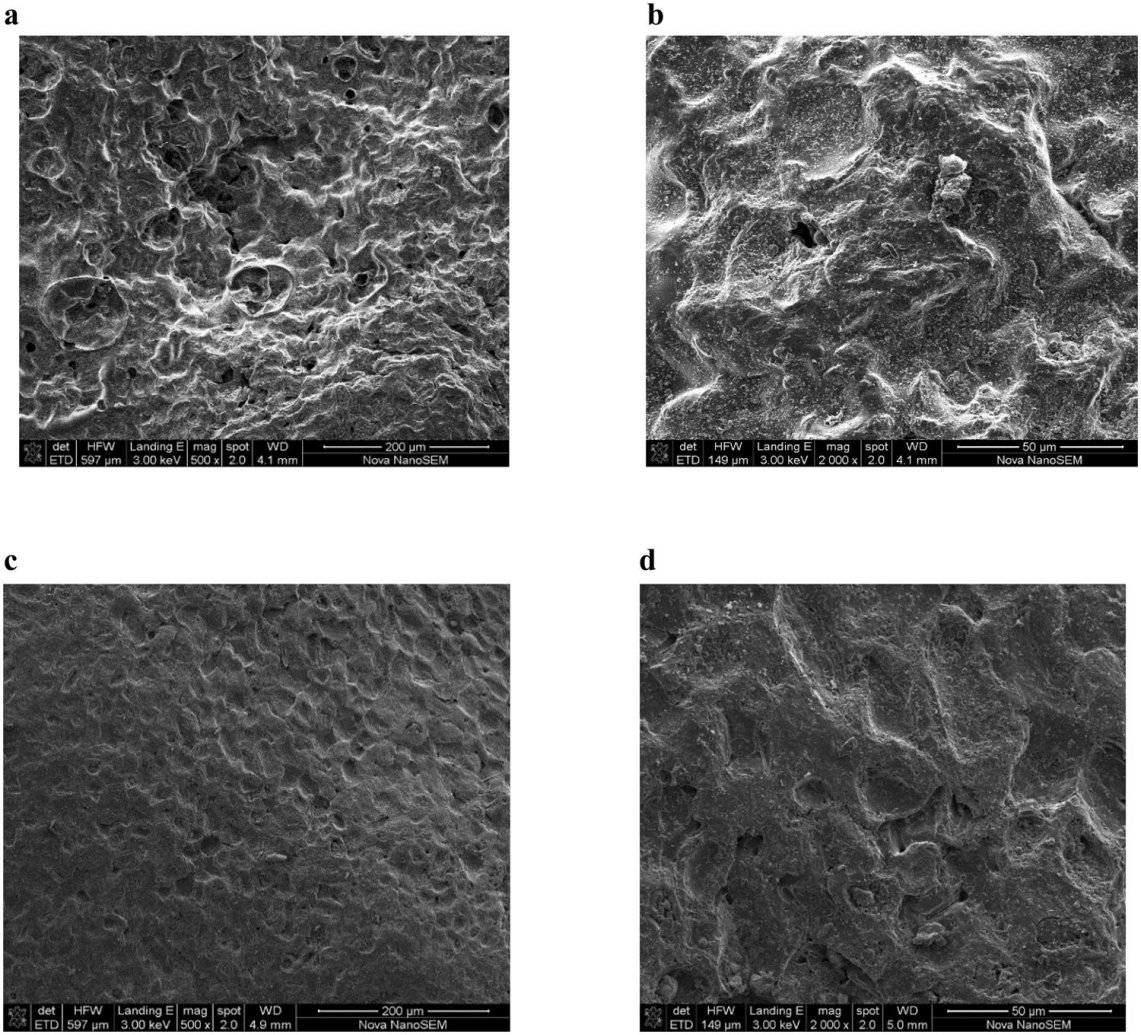
Scanning electron microscopy of ZnCl_2_-treacted carbon activated at 300 °C magnified at (**a**) 500× and (**b**) 2000× respectively; carbon activated at 400 °C, magnified at (**c**) 500× and (**d**) 2000× respectively.

SEM characterization is typically carried out to study surface morphologies of materials. The effect of chemical agents and temperature on the textural surface morphology of the activated carbon is shown in Figs 3 and 4. Compared to the activated carbon pyrolysed at 300 °C, the SEM photographs for H_3_PO_4_-activated carbon pyrolysed at 400 °C showed non-uniform pore size distribution, probably arising from rapid pore development resulting in formation of larger cavities and cracks. However ZnCl_2_-activated carbon showed a smoother surface with numerous small pores. Furthermore, H_3_PO_4_ clearly produced larger cavities at higher temperature whereas for ZnCl_2_ activation, the surface was smoother at higher temperature.

#### FT-IR and BET Characterization

**Figure 5:**
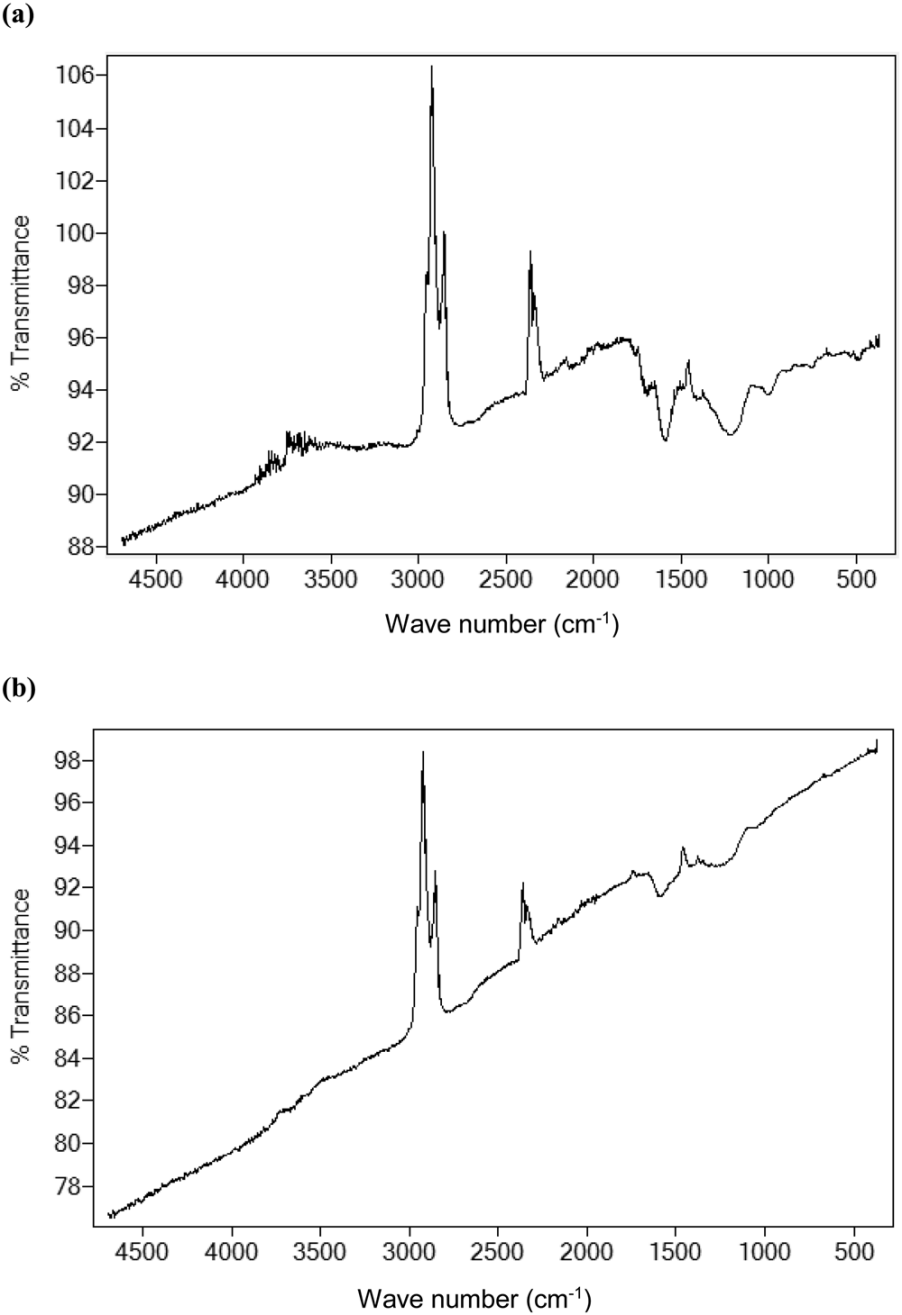
(**a)** FT-IR Spectrum of H_3_PO_4_ treated carbon activated at 300 °C, **(b)** FT-IR Spectrum of H_3_PO_4_ treated carbon activated at 800 °C.

FT-IR spectra obtained on the study of how the functional groups on granular activated carbon changed with increasing temperature are shown in Fig. 5. For the granular activated carbon produced at 300 °C, the weak peak observed at 1702.1 can be assigned to an unsaturated C=O group, strong peak at 1600.85 to an aromatic C=C group, band centred at 1215.85 to phenol or acyl group, and the weak peak at 1004.24 can be assigned to an alkoxy group. The band at ca. 1601 can be assigned to aromatic C=C, whilst the band at ca. 1214 is possibly due to a phenol or acyl C-O group. BET surface areas of 503 and 805 m^2^/g were obtained for H_3_PO_4_ and ZnCl_2_-treated granular activated carbons, respectively.

#### Effect of activated carbon dose

**Figure 6:**
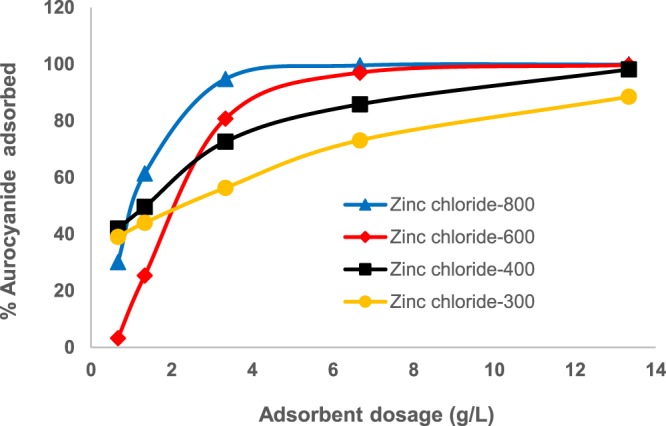
Effect of carbon dosage on aurocyanide adsorption (Initial concentration of 20.85 mg/L, pH 10, contact time of 24 h, 100 rpm agitation rate).

Plots for Zinc chloride-600 and Zinc chloride-800 shown in Fig. 6 gave optimum adsorbent concentrations of 7.0 and 4.0 g/L corresponding to 97 and 98% aurocyanide uptake, respectively. The saturation curves for Zinc chloride-300 and Zinc chloride-400 were not sharply defined in the adsorbent range under study, making it difficult to deduce the optimum adsorbent concentration.

#### Equilibrium modeling

The Langmuir adsorption isotherm, which is probably the most widely applied isotherm, was used to analyze the sorption data. For monolayer saturation, the Langmuir isotherm can be represented as:

6 $${q}_{e}=\frac{b{q}_{m}{C}_{e}}{1+b{C}_{e}}$$

and linearized as:

7 $$\frac{{C}_{e}}{{q}_{e}}=\frac{1}{{q}_{m}b}+\frac{{C}_{e}}{{q}_{m}}$$

where *C*_*e*_ is the equilibrium concentration (mg/L), *q*_*e*_ is the amount of metal ion adsorbed (mg/g), *q*_*m*_ is the *q*_*e*_ for a complete monolayer (mg/g) and *b* is a constant related to the effect of the binding sites and the energy of the adsorption (L/mg).

**Figure 7:**
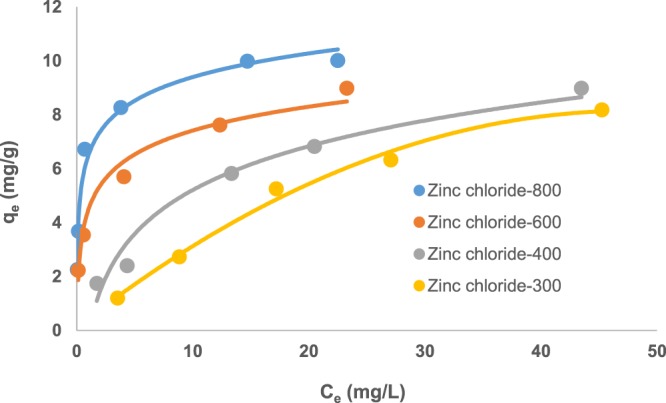
Comparison of the experimental isotherms for aurocyanide adsorption (pH 10, adsorbent dose of 3.3 g/L, contact time of 24 h, 100 rpm agitation rate).

**Figure 8:**
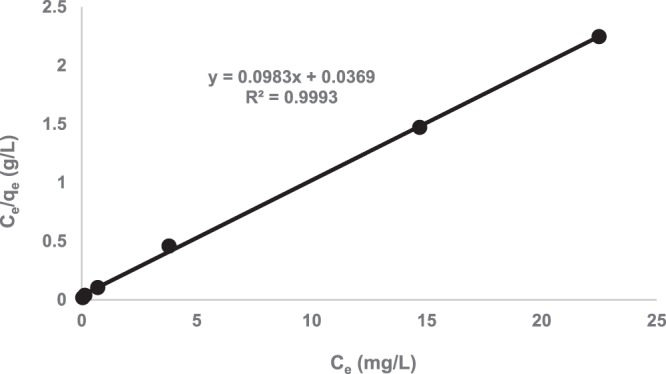
Langmuir isotherm plot for Zinc chloride-800 (pH 10, adsorbent dose of 3.3 g/L, contact time of 24 h, 100 rpm agitation rate).

Figure 7 shows the experimental isotherm data for Zinc chloride-treated carbon activated at temperatures ranging from 300–800 °C. The results showed that Zinc chloride-800 gave the highest experimental adsorption capacity (*q*_*e*_ = 10 mg/g), and a *q*_*m*_ value of 13.1 mg/g (obtained using the linearized Langmuir plot shown in Fig. 8).

#### Kinetic modeling

In modelling industrial reactors, it is important to consider kinetics and equilibrium properties of processes. It is necessary to predict how much time is required to reach equilibrium and the rate at which reactions occur in order to predict the optimum conditions for the processes. In adsorption studies, kinetics is expressed as the solute removal rate that controls the residence time of the sorbate in the solid-solution interface. The adsorption data obtained in the current study were modelled using the first order, second order, pseudo second order and Elovich kinetic equations shown in Equations (8–11).

8 $$ln{C}_{t}=-{k}_{1}t+ln{C}_{0}$$

9 $$\frac{1}{{C}_{t}}={k}_{2}t+\frac{1}{{C}_{0}}$$

10 $$\frac{t}{{q}_{t}}=\frac{1}{({k}_{3}\ast {q}_{e}^{2})}+\frac{t}{{q}_{e}}$$

11 $${q}_{t}={K}_{id}{t}^{0.5}+C$$

where *C*_0_ is the initial concentration of aurocyanide (mg/L), *C*_*t*_ is the concentration of aurocyanide after time *t* (mg/L), *k*_1_, *k*_2_, *k*_3_, ${K}_{id}$ are the rate constants for first order adsorption, second order adsorption, pseudo-second order adsorption, and intra-particle diffusion, respectively.

**Figure 9:**
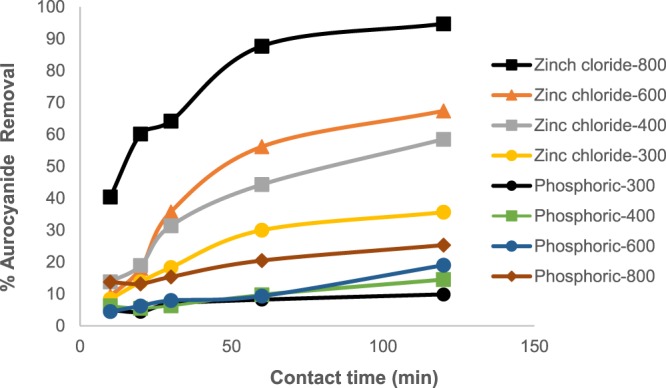
% Removal of aurocyanide against contact time.

**Figure 10:**
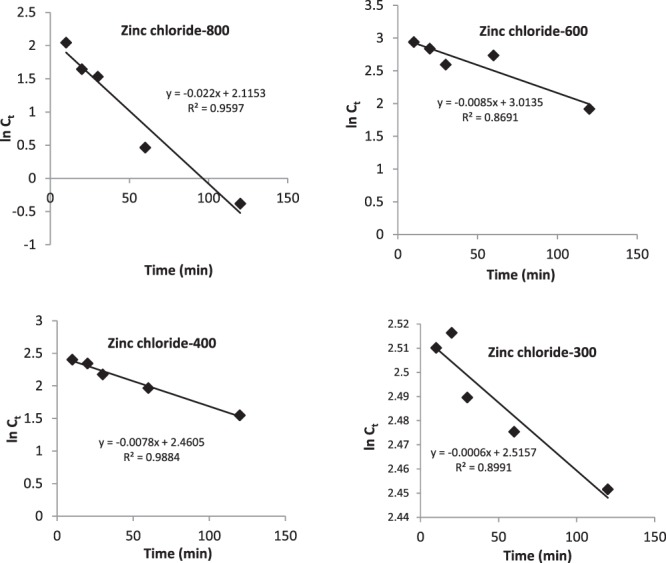
First order kinetic model for adsorption of aurocyanide by carbon activated under various conditions.

**Figure 11:**
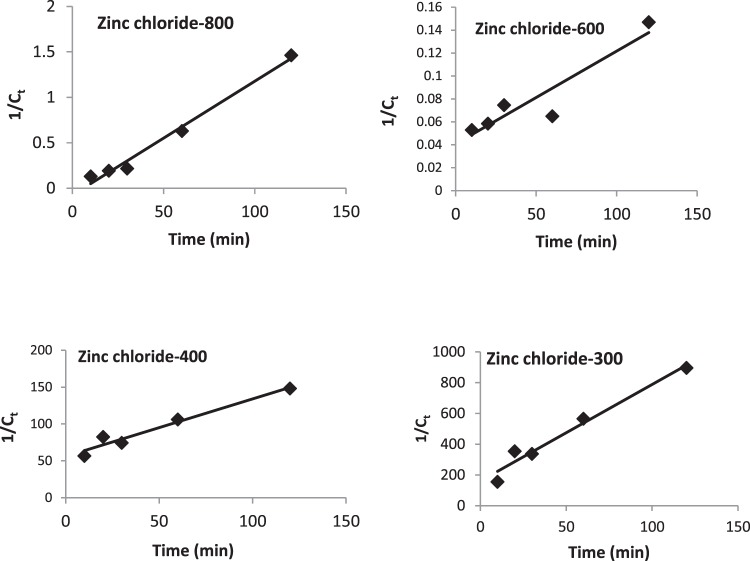
Second order kinetic model for adsorption of aurocyanide by carbon activated under various conditions.

**Figure 12:**
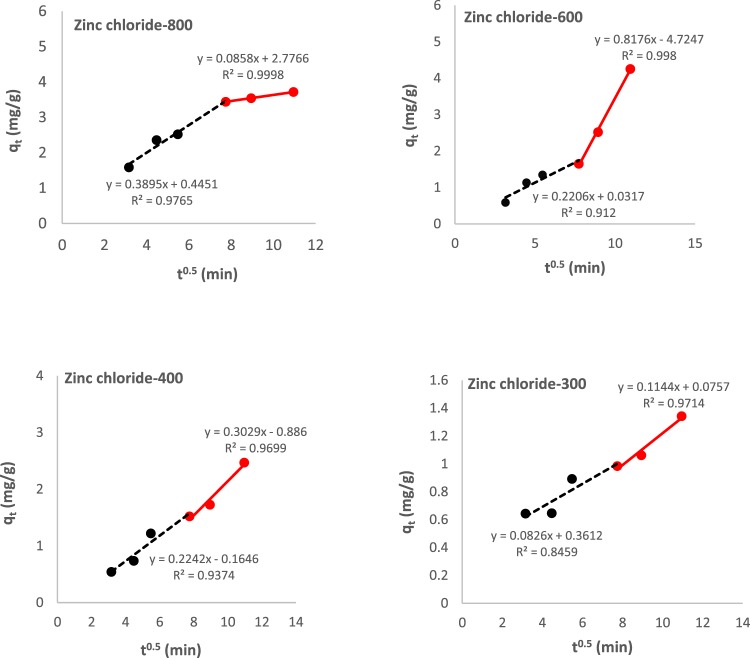
Plot of *q*_*t*_ vs. ${t}^{0.5}$ for intra-particle diffusion model.

Figures 9–12 show graphs for the kinetic analysis of aurocyanide adsorption data and the kinetic parameters obtained are shown in Table 1. In Fig. 9, the graphs of % aurocyanide removal from solution against contact time for various heat and chemically-activated carbons show that ZnCl_2_ impregnated samples have higher % removal than H_3_PO_4_ treated ones, achieving close to 100% recovery for ZnCl_2_ treated activated at 800 °C in 2 h.

**Table 1:** The comparison of first order, second order, and pseudo second order adsorption rate constants.

Sample	First order		Second order		Pseudo second order	
	*k*_1_	*R* ^2^	*k*_2_	*R* ^2^	*k*_3_	*R* ^2^
Zinc chloride-800	0.022 ± 0.0008	0.9597	0.0125 ± 0.002	00.9862	0.0412 ± 0.002	0.9972
Zinc chloride-600	0.008 ± 0.0009	0.8691	0.008 ± 0.005	0.8673	0.00367 ± 0.0005	0.4156
Zinc chloride-400	0.007 ± 0.0003	0.9884	0.7793 ± 0.003	0.9592	0.0108 ± 0.004	0.9592
Zinc chloride-300	0.001 ± 0.0002	0.8991	6.2738 ± 0.06	0.9674	0.245 ± 0.003	0.9674

## Discussion

Use of chemical agents resulted in reduced loss of precursor material, with H_3_PO_4_ activation giving much lower % Burn-off. The amount of material lost during the physical activation step followed the order Control > Zinc chloride > Phosphoric acid-activated carbon. However, the % Hardness surprisingly also followed the same order. We would have expected that increase in material hardness would translate into reduction in % Burn-off. This shows that although the use of chemical agents minimized loss of material during activation, the same agents reduced the hardness of the material. It was also observed that the values of % Burn-off observed in this study (50–84%) were comparable to those reported previously^23^. The range in % Burn-off values reported for different precursor materials has been explained in terms of the loss of volatile matter associated with the decomposition of compounds such as lignin, hemicellulose, pectin, and cellulose^24^. Naturally, the relative amounts of these compounds vary with precursor material and hence the differences in the % Burn-off reported by various authors, even for the same temperature range and activation chemicals^25^. It is also clear that ZnCl_2_-activated carbon showed higher % Hardness number compared to the samples produced using H_3_PO_4_ as the activation agent. These findings could be explained by considering Zinc chloride to be acting as a Lewis acid during pyrolysis reactions, leading to evolution of molecular hydrogen and subsequent polymerization (aromatization) of the remaining adjacent molecules, resulting in a stable structure^26^. It can be mentioned that although the % Hardness number is a measure of resistance to crushing and breakage of granular carbons, it is only reliable when comparing carbons of the same form. Due to plant to plant variations in practical gold recovery operations, it is extremely difficult to verify the predictive value of any type of laboratory hardness tests on absolute plant carbon losses or even relative plant operations.

Compared to granular activated carbon produced at 300 °C, the SEM photographs for H_3_PO_4_-activated carbon pyrolysed at 400 °C showed non-uniform pore size distribution, probably arising from rapid pore development resulting in formation of larger cavities and cracks. However ZnCl_2_-activated carbon showed a smoother surface with numerous small pores. Furthermore, H_3_PO_4_ activation clearly produced larger cavities at higher temperature whereas for ZnCl_2_ activation, the surface was smoother at higher temperature. In related studies, Demiral *et al*.^27^ reported that treatment of hazelnut bagasse with ZnCl_2_ produced activated carbon full of cavities while the samples activated with KOH had smoother surfaces. Furthermore, Timur *et al*.^28^ found that when using oak cups pulp as precursor, ZnCl_2_ activated carbons were full of cavities while for H_3_PO_4_ activated carbons, the external structure was intact. It was also reported that chemical treatment of palm kernel shells produced highly defined pore cavities for ZnCl_2_, while for K_2_CO_3_, the surface was non-uniform^29^. In the current study, loss of volatile compounds is suggested by the FT-IR spectra, which showed that the number of bands decreased with increasing temperature, from 5 bands discernable at 300 °C to 2 bands at 800 °C. Furthermore, presence of the aromatic ring or C=C indicated formation of carbonyl-containing groups and aromatization of the precursor during pyrolysis. Other authors have proposed that mechanisms involved during the use of different activating chemicals were different. For instance, it was proposed that the use of ZnCl_2_ could promote loss of water and/or molecular hydrogen from the lignocellulosic structures of the parent materials or the precursors, whereas H_3_PO_4_ could combine chemically within the lignocellulosic structures^25,30^.

The motivation to study effect of adsorbent dose on uptake of a sorbate from solution is mainly economic, i.e., to establish the optimum concentration of the adsorbent which could lead to a maximum adsorption performance for a given system^31,32^. Increasing adsorbent concentration should result in increased percentage adsorption performance saturating at a certain adsorbent dosage level characteristic of the adsorption system. Usually, the amount of solute remaining in solution decreases with increasing adsorbent dose as more adsorption sites become available. In the current study, optimum adsorbent concentrations of 4.0 and 7.0 g/L were obtained for Zinc chloride-800 (98% aurocyanide uptake) and Zinc chloride-600 (97% aurocyanide uptake), respectively. These optimized adsorbent concentration values were higher than those reported for adsorption of aurocyanide (87%), using activated carbon produced from coconut shells as precursor (1.25 g/L)^2^. Furthermore, the findings suggest that higher temperatures produced activated carbon which was more effective in the adsorption of aurocyanide. These conclusions were corroborated by equilibrium modeling, which showed that higher *q*_*e*_ values were obtained for carbons pyrolized at higher activation temperatures. It can be concluded that Zinc chloride-activated carbon achieved higher *q*_*e*_ values compared to the H_3_PO_4_-acid treated carbon, and that ZnCl_2_-800 was the most effective adsorbent for the uptake of aurocyanide from solution. These findings are consistent with the higher BET surface area obtained for ZnCl_2_-treated samples compared to the H_3_PO_4_-treated ones. It can also be concluded that although higher activation temperatures resulted in lower yields of activated carbon, the carbons produced at such temperatures were more effective for aurocyanide adsorption. This could be explained in terms of increased surface area, total pore volume, and porosity achieved at higher temperature^25^. The higher aurocyanide uptake recorded for ZnCl_2_-treated granular activated carbon when compared to H_3_PO_4_-treated carbon could be explained in terms of the numerous fine pores (hence larger surface area) observed for the ZnCl_2_-treated carbon as confirmed by the SEM images.

Equilibrium modeling of the sorption data showed that showed that Zinc chloride-800 gave the highest experimental adsorption capacity (*q*_*e*_ = 10 mg/g), and a *q*_*m*_ value of 13.1 mg/g. These values are higher than those reported for aurocyanide adsorption using coconut shell-derived carbon (*q*_*m*_ = 1.79 mg/g)^2^, and surface functionalized biomass (*q*_*e*_ = 7.97 mg/g)^33^. Further comparison showed that the equilibrium adsorption capacity values obtained in this study were also higher than those reported for aurocyanide adsorption using activated carbon derived from hard shell of apricot stones (6.03 mg/g at 25 °C) but similar to those obtained at 40 °C (12.01 mg/g)^34^.

From kinetic studies, it was shown that >95% of aurocyanide was recovered in 120 min from solution using ZnCl_2_ treated granular carbon activated at 800 °C. This was higher than values obtained for activated carbon derived from coconut shell^2^. Further analysis showed that the aurocyanide sorption data was adequately described by the pseudo-second order model (R^2^ > 0.99). Plots of *q*_*t*_ vs. ${t}^{0.5}$ obtained for intra-particle diffusion kinetic model shows that the intercepting lines do not pass through the origin (i.e., C≠0), thus suggesting that intra-particle diffusion was not the rate determining step^33^. Furthermore, multi-linearity is clearly evident for Zinc chloride-800 and Zinc chloride-600. It is also interesting to note that for Zinc chloride-800, the intra-particle diffusion model plot shows that the aurocyanide adsorption process was controlled by a combination of external mass transfer (initially represented by the steeper slope: gradient of 0.390 mg/g.min^0.5^) and intra-particle diffusion represented by a less-steeper slope (gradient of 0.0858 mg/g.min^0.5^)^33^. The value of intra-particle diffusion rate constant obtained for Zinc chloride-800 (*k*_*id*_ = 8.58 × 10^−2^ mg g^−1^ min^0.5^) was comparable to the rate constant reported for aurocyanide adsorption using coconut shell-derived activated carbon (8.48 × 10^−2^ mg g^−1^ min^0.5^)^2^. However, the mechanistic steps appear to be reversed for Zinc chloride-600, suggesting that intra-particle diffusion is more significant during the initial phase of adsorption before mass transfer effects become more pronounced. These observations are consistent with findings from surface characterization, e.g., for Zinc chloride-800, the higher surface area reported implies that once the solute particles reached the adsorbent, adequate surface area was available for adsorption and therefore overall rate of adsorption was limited by the rate at which the solute particles reached the surface, i.e., by mass transfer effects. In conclusion, varying activation temperature resulted in adsorbents showing different mechanistic properties for aurocyanide uptake.

Future studies should seek to improve % Hardness of the granular activated carbon and reduce its % Burn-off. One way is to use carefully selected binders, which do not negatively alter adsorption properties of the material. There is also need to further investigate how varying the duration of activation process, activating agents, and agent-precursor impregnation ratio, would affect the physico-chemical properties of the activated carbon produced.

Our study demonstrates that granular activated carbon derived from peach stones as precursor can effectively remove aurocyanide from aqueous solution. The findings provide a typical example of how value can be unlocked from agricultural wastes. In addition, our study suggests that the appropriate selection of activating chemical agents coupled with appropriate activation temperature can produce granular activated carbon with potential for development as alternative adsorbent for aurocyanide.

## Methods

### Instrumentation

The carbonisation experiments were carried out in a locally constructed horizontal furnace shown in Fig. 10. Scanning electron microscopy (SEM) morphologies were done in an FEI Nova Nano SEM 230 instrument (Holand) and images were acquired at magnification of 500×, 2000×, and 10000× at University of Cape Town, SA. IR studies were carried out using a Perkin-Elmer Spectrum 100 FT-IR over a range of 4000–4500 cm^−1^. Specific surface area was determined according to the Brunaru Emmet-Teller (BET) protocol on a Micromeritics model ASAP 2000. Aurocyanide solution was determined using a Variation Atomic Absorption Spectrophotometer at the Institute of Mining Research at the University of Zimbabwe.

### Reagents and materials

All chemicals and reagents used in this work were of analytical grade and purchased from Sigma Aldrich, Germany. The water used for aqueous phase was MiliQ water (A Grade) for all experiments.

### Preparation of peach stones

Peach stones collected from jam-manufacturing companies were cleaned thoroughly with water to remove the remnants of peach flesh from the stones. The stones were thoroughly washed with tap water until the previously attached material was removed. They were then rinsed with distilled water and dried in the sun. The stones were then crushed with a roller crusher at the Institute of Mining Research at University of Zimbabwe to remove the seeds inside. The remaining stones were then washed again with distilled water and air dried in the sun. The dry stones were subsequently put into a jaw crusher to get granules. The granules obtained were then sieved through the mesh wire to obtain the 8 × 16 size fraction or 1.7 mm mean particle size of peach stones. The obtained peach stones were then subjected to different carbonisation and activation processes.

### H_3_PO_4_ Activation

Raw peach stones (100 g) were impregnated with 43% (wt) H_3_PO_4_ for 24 hours at 85 °C in weight ratio 1:1 (H_3_PO_4_: peach stones), ensuring that they were completely immersed in H_3_PO_4_ to prevent oxidation. This was followed by drying in the oven overnight at 105 °C. The dried sample of impregnated peach stones was then put into the activating furnace at 300–800 °C temperature for 1 h 20 min under a nitrogen environment. The sample was allowed to cool and subsequently washed first in hot sodium hydroxide solution to neutralize excess H_3_PO_4_, followed by a series of soaking and decanting in distilled water until there were no traces of phosphates (identified by adding a few drops of Pb(NO_3_)_2_ into the wash water).

### ZnCl_2_ Activation

Zinc chloride (3.3 g) was dissolved in 100 ml distilled water. Raw peach stones (100 g) were then soaked in this solution overnight at 85 °C for 24 h. At the end of this period, the solution was filtered and the impregnated peach stones were dried in an oven overnight at 105 °C and then activated by heating in the activating furnace at 300–800 °C for 1 h 20 min under nitrogen environment and allowed to cool. The sample was washed in hot HCl solution at 2 M concentration and rinsed in distilled water until the wash water was around pH 3. The control samples were prepared by subjecting the raw peach stone samples to the activating furnace at 300–800 °C for 1 h 20 min without chemical activation and allowed to cool.

### Carbonisation Furnace

**Figure 13:**
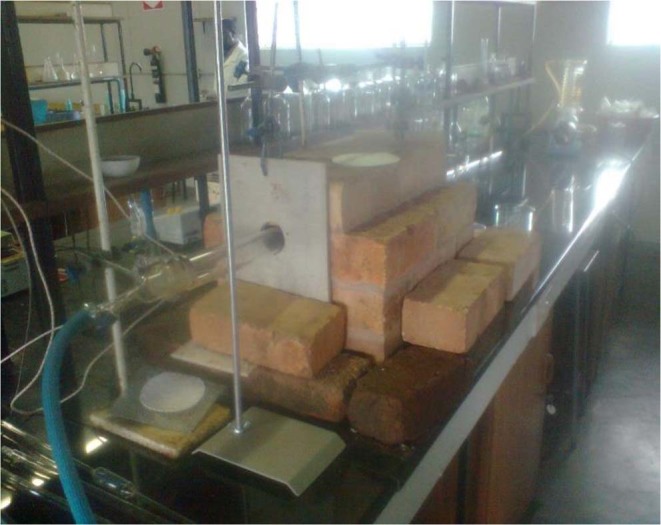
Locally assembled carbonization furnace.

The carbonisation experiments were carried out in a locally constructed horizontal furnace shown in Fig. 13. To ensure an inert furnace atmosphere, nitrogen gas flow was introduced into the 40 mm diameter quartz tube of length one metre which was placed into the furnace. The inlet and output of the quartz tube were connected to pyrex glass. The furnace was constructed by using fire brick and Nichrome wire as the heating element. This was then covered with electrician’s clay to prevent oxidation of the element by air. A temperature probe was then inserted just outside the clay covering. The temperature controller was then incorporated into the circuit to regulate temperatures within the programmed range.

### Carbonisation Process

Samples of granular peach stones (100 g) were placed in the locally assembled furnace (Fig. 13). Nitrogen (supplied by BOC gases, Zimbabwe) was released to purge the air inside the quartz tube for 30 min. After purging, the furnace was switched on to heat till the desired carbonisation temperature was achieved and pyrolysis allowed to continue for a predetermined time. After heating, the sample was cooled by nitrogen gas until temperatures below 100 °C were reached.

### Determination of physical and chemical properties

#### Burn-off and Carbon Yield

Carbon burn-off is the weight loss of the pyrolysed carbon, determined on a dry weight basis that occurs during the activation process^23^. The % burn-off was calculated by recording the weight of dry peach stone before and after activation. The procedure was repeated 3 times.

#### Hardness Number

Hardness determinations were carried out as previously described^30^. Three grams of granular carbon were placed in a 250 ml Erlenmeyer flask. Ten glass marbles of about 5 g each were placed into the flask, which was then continuously agitated at 200 rpm in a water bath shaker for 15 min at 25 °C.

### Adsorption studies

#### Preparation of gold solution (adsorbate)

A borate buffer solution to control pH was prepared by mixing boric acid (H_3_BO_3_) at 3.101 g/L, calcium chloride (CaCl_2_.H_2_O) at 3.221 g/L, potassium cyanide (KCN) at 0.0027 g/L. The buffer solution was adjusted to approximately pH 10. To this background a standard solution of 500 ppm Au in the form of Au(CN)_2_^−^ was used to make up the solutions in all experiments. A mass of 0.747 g of KAu(CN)_2_ was weighed and made up to 1 L volumetric flask using distilled water to produce the standard solution.

#### Effect of carbon dosage

Activated carbon samples (0.1–2.0 g) were added to aurocyanide solution (10 mg L^−1^, 150 mL), pH 10, and agitated at 100 rpm for 24 h at room temperature. The mixture was subsequently filtered to remove suspended solids and the filtrate analyzed for gold concentration using Flame Atomic Absorption Spectrometry. The same procedure was repeated 3 times to obtain mean values for the measurements.

#### Kinetic studies

To find the effect of contact time on aurocyanide adsorption from solution using activated carbon samples, 1.5 g of activated carbon was added to each of five 500 mL conical flasks. One hundred and fifty milliliters of the aurocyanide solution of concentration 10 mg/L was added followed by agitation of the mixture at 100 rpm using a KAHN shaker. After agitation for a specified contact time (10–120 min), each sample was immediately filtered to separate the adsorbate from the carbon particles. The filtrate was analysed to determine the concentration of the aurocyanide using Flame AAS. The same procedure was repeated 3 times to obtain a mean value for the measurements.

## Data Availability

Data is available and will be uploaded.
